# Editorial: Advancing drug discovery with AI: drug–target interactions, mechanisms of action, and screening

**DOI:** 10.3389/fphar.2025.1721323

**Published:** 2025-10-24

**Authors:** Xutong Li, Jing Xing, Sulin Zhang, Jiayu Zhou

**Affiliations:** ^1^ Shanghai Institute of Materia Medica, Chinese Academy of Sciences, Shanghai, China; ^2^ Lingang Laboratory, Shanghai, China; ^3^ School of lnformation, University of Michigan, Ann Arbor, MI, United States

**Keywords:** drug–target interactions, mechanisms of action, screening, drug discovery, artificial intelligence, molecular docking and dynamics simulations, omics

## Introduction

Computer-aided drug design (CADD) began as a physics- and knowledge-driven discipline: docking, QSAR, pharmacophore modeling, and molecular dynamics (MD) provided a rational scaffold for hit finding and lead optimization. These tools excelled at exploring how candidate molecules might bind, but they were limited by library size, scoring biases, and a narrow view of biological context. The past decade has added a second, data-centric layer: machine learning and deep learning have scaled pattern discovery across chemical and biological spaces, elevated generalization from local heuristics to global signals, and enabled generative design. In parallel, target identification—the upstream question of *where* to intervene—has increasingly shifted from single-gene hypotheses to AI-assisted hypothesis generation over networks, knowledge graphs, and large unlabeled corpora. Most recently, a third layer has matured: omics-anchored pharmacology, in which transcriptomic, proteomic, and interactome signals ground mechanism-of-action (MoA) inference, drug repurposing, and patient stratification.

This Research Topic brings together a diverse set of contributions that highlight recent advances in AI-driven pharmacology. The collected works include (i) applications that connect AI/CADD workflows to therapeutic challenges in oncology, antivirals, antifungals, traditional formula optimization, and combination therapy, and (ii) algorithmic advances that expand representation learning for drug–target interactions (DTIs) and drug synergy modeling. Collectively, these studies showcase how AI can reshape both the theoretical and translational dimensions of modern drug discovery.

## Computer-aided drug design (CADD) as the foundation

As the historical foundation of computer-aided drug discovery, structure-based methods remain indispensable for rational hit identification and validation. Zong et al. demonstrated a structure-based workflow for discovering inhibitors of the SARS-CoV-2 main protease (3CLpro) (Zong et al.). Their study combined docking, *in vitro* enzymatic assays, MD simulations, and density functional theory analyses to identify low-micromolar inhibitors and to characterize their binding dynamics. This work exemplifies a classical CADD paradigm applied to an urgent antiviral target, highlighting the enduring value of structure-based modeling reinforced by experimental validation.

While Zong et al. exemplified a purely structure-based pipeline, Sun et al. showed how deep learning can extend this paradigm toward generative exploration of chemical space (Sun et al.). They developed a pipeline for *de novo* design against mutant IDH1 (mIDH1) that integrated a bidirectional recurrent neural network with scaffold hopping. The resulting molecular candidates were evaluated through ADME prediction, docking, and MD simulations. This approach illustrates the transition from physics-driven CADD to AI-driven generative design, blending data-centric exploration with rigorous structural checks.

## AI for drug–target interaction discovery at scale

Where CADD refines *how* molecules bind, DTI modeling addresses *to whom* they bind. Deng et al. introduced GNNBlockDTI, a substructure-aware graph neural network that organizes multiple GNN layers into functional “blocks” (Deng et al.). Each block captures drug substructures at different levels of granularity, from local motifs to global topology. To represent proteins, they designed a local encoding strategy that emphasizes pocket-level features, closely mimicking the binding environment. Feature enhancement and gating mechanisms further reduced redundancy and noise.

Sun advanced the field with a multimodal learning framework capable of integrating molecular graphs, protein sequences, transcriptomic data, textual descriptions, and bioassay information (Sun). Using a hierarchical attention fusion strategy, the Unified Multimodal Molecule Encoder (UMME) aligned intra- and inter-modal representations. To handle incomplete or noisy inputs, Sun introduced Adaptive Curriculum-guided Modality Optimization (ACMO), which prioritizes reliable modalities during early training and gradually incorporates uncertain ones.

Ge et al. shifted the focus from single drug–target pairs to drug–drug synergy prediction, presenting the MD-Syn framework. This model integrates one-dimensional features (SMILES-based embeddings and cell-line expression profiles) with two-dimensional features (molecular graphs and protein–protein interaction networks) (Ge et al.). A multi-head attention mechanism highlights the most influential feature aspects, thereby improving interpretability. Importantly, the team released a public web server, enabling the broader community to predict synergy effects with custom compounds. This effort demonstrates how methodological advances can translate into practical, user-oriented tools.

Together, these studies show how DTI research is progressing from unimodal descriptors to multimodal integration, balancing methodological innovation with translational accessibility.

## AI with omics for mechanism and translation

Accurately mapping drug–target interactions is fundamental for understanding therapeutic specificity and polypharmacology, and AI innovations are now scaling this task to increasingly complex biological contexts. Chai et al. identified Dp44mT as an antifungal candidate through ensemble learning, and validated its activity with transcriptomic and proteomic profiling, mitochondrial assays, and MD simulations (Chai et al.). Their findings revealed that disruption of cellular iron homeostasis induces mitochondrial dysfunction and apoptosis in *Candida albicans*. This closed-loop workflow—from AI prioritization to omics-guided mechanistic validation—illustrates the maturation of integrative pharmacology.

He et al. focused on optimizing the traditional Chinese medicine “eczema mixture” (He et al.). By combining UPLC-Q/TOF chemical profiling with a back-propagation neural network and a multi-objective evolutionary algorithm, they identified compatibility patterns and dosage ratios that enhanced therapeutic efficacy. Key roles were confirmed for *Phellodendron chinense* and *Sophora flavescens*, providing mechanistic rationale for classical prescriptions. This study shows how AI can bring quantitative precision to complex multi-component therapies, bridging traditional knowledge with modern pharmacology.

Together, by grounding predictions in biological pathways and experimental data, these studies move beyond statistical associations to causal insights, paving the way for more reliable and translatable therapeutic innovations.

## Future directions

From this Research Topic, three cross-cutting priorities emerge. First, multimodal and multi-scale integration: the most effective models combine chemical structure, protein context, and cellular state, while treating missing data as a norm rather than an exception. Second, mechanistic plausibility and translation: linking AI predictions to MD/DFT simulations, omics readouts, or perturbational assays ensures interpretability and reduces experimental risk. Third, human-centered usability: open platforms, interpretable attention maps, and optimization frameworks for combinations or polyherbal medicines transform algorithms into practical decision-support tools.

Beyond these themes, emerging therapeutic modalities represent an important frontier. Zhang et al. reviewed the progress of peptide–drug conjugates (PDCs), which combine the specificity of peptides with the potency of small molecules (Zhang et al.). They highlighted challenges such as limited peptide libraries and linker chemistries, and emphasized opportunities where AI can accelerate peptide selection, linker optimization, and therapeutic evaluation. This perspective underscores how AI is broadening its scope from small molecules to new therapeutic formats, expanding the horizon of precision pharmacology.

As Research Topic Editors, we see these contributions as a coherent step toward AI systems that are generative, grounded, and generalizable—systems that not only explore chemical space but also reason over targets and mechanisms, and that integrate omics evidence to close the loop between computation and experiment. Harnessing this triad will help deliver safer, faster, and more precise therapeutics.

